# A terpenoid isolated from sarang semut (*Myrmecodia pendans*) bulb and its potential for the inhibition and eradication of *Streptococcus mutans* biofilm

**DOI:** 10.1186/s12906-018-2213-x

**Published:** 2018-05-09

**Authors:** Meirina Gartika, Hening T. Pramesti, Dikdik Kurnia, Mieke H. Satari

**Affiliations:** 1 Department of Oral Biology, Faculty of Dentistry, Universitas Padjadjaran, Bandung, Indonesia; 2 Department of Chemistry, Faculty of Mathematic and Natural Sciences, Universitas Padjadjaran, Bandung, Indonesia; 3 Department of Pediatric Dentistry, Faculty of Dentistry, Universitas Padjadjaran, Bandung, Indonesia

**Keywords:** Streptococcus *mutans* biofilm, Terpenoid, *Myrmecodia pendans*

## Abstract

**Background:**

Dental caries remains a serious problem due to its detrimental effects on individual health and quality of life. The bulbs of *Myrmecodia pendans* (Merr & Perry), native plants of Papua, have been used as natural remedies for tumours, gout, diarrhoea, and fever. In this study, one of the active compounds of *M. pendans* was isolated, and its biological activity against the formation of *Streptococcus mutans* ATCC 25175 biofilm was tested.

**Methods:**

*M. pendans* was extracted with ethyl acetate using a Soxhlet apparatus. The extract was then separated, and chromatographic purification provided the isolated compound. The structure of the active compound was then characterized using UV, IR, NMR, and MS spectrometry. The obtained compound was added to *S. mutans* biofilms to determine the MBIC and MBEC values.

**Results:**

The compound isolated from *M. pendans* was determined to be a labdane diterpene derivative with the formula C_31_H_50_O_3_. The MBIC value of the terpenoid towards the *S. mutans* biofilms was 50 ppm, and the MBEC value for the 1 min induction time was 40%.

**Conclusion:**

The terpenoid extracted from *M. pendans* has the potential to be developed into an antibacterial agent particularly for preventing the formation of biofilms.

## Background

Dental caries remains a serious problem because it can trigger further health problems. Dental caries is a multifactorial disease. Interactions between cariogenic agents will impact the health of the tooth due to the presence of cariogenic biofilms on the tooth surface [1]. *Streptococcus mutans* is considered one of the cariogenic bacteria due to its ability to form biofilms [2, 3].

Currently, caries prevention is aimed at preventing the formation of dental biofilms or reducing the amount of *S. mutans* in the biofilms [3]. Some chemical compounds are commonly used in many dental products to suppress the growth of the bacteria, but chemical compounds often cause many side effects [4, 5]. Therefore, many studies have been conducted on the use of natural compounds for the prevention of disease.

*Myrmecophytes (Myrmecodia pendans*, literally “ant-plant”) originates from the Papua Islands, which are located in the eastern part of Indonesia. This plant can also be found in the Malay Peninsula, the Philippines, Cambodia, Sumatera, Java, Cape York, and the Solomon Islands. *M. pendans* belongs to the *Rubiaceae* family, which contains five genera, and only two of these genera are associated with ants, *Myrmecodia* (45 species) and *Hydnophytum* (26 species). Of all these species, only *H. formicarum*, *M. pendans*, and *M. bulbosa* are often used as natural remedies. *M. pendans*, known by the Papuans as a medicinal plant, can be used to treat various diseases, such as cancer, tumours, gout, diarrhoea, and fever [6, 7]. Soeksmanto’s research on rats showed that the toxicity of *M. pendans* extract at a dose of 375 mg/kg bw caused liver degeneration, whereas a dose of 3.750 mg/kg bw caused cell necrosis [8].

## Methods

### Chemicals and reagents

Silica gel resin 60 Li Chroprep RP-18 (Merck®), Kiesel gel 60 F254 and RP-18 F254S were purchased from Merck® (Darmstadt, Germany). Some of the solvents used in this study were purchased from Merck Co. Ltd., and the rest were purchased from Sigma Aldrich Co. Ltd. (St. Louis, MO, USA).

### Plant material collection and determination

Dried bulbs of *M. pendans* were acquired from Papua and were identified by Mr. Joko (Botanist) at the Laboratory of Plant Taxonomy, Department of Biology, Faculty of Mathematics and Natural Sciences Universitas Padjadjaran, Bandung, Indonesia.

### Extraction of the bulbs of *M. pendans*

The extraction of *M. pendans* bulbs was performed using a Soxhlet apparatus because the associated procedures are relatively fast and require less solvent than maceration methods [9]. Additionally, based on prior research on the isolation of these compounds, a temperature of 40 °C was used to prevent the decomposition of the compounds in the extract. Heating at 40 °C during the Soxhlet extraction process will not damage the constituents; therefore, the compounds contained in the *M. pendans* bulb were thermally stable. Ethyl acetate was used in the extraction process because after conducting a qualitative test with a thin-layer chromatography, the target compound was found in the ethyl acetate fraction.

As much as 1.5 kg of *M. pendans* bulbs was cut into small pieces to extract the maximum amount of each compound. Bulb pieces were extracted with as much as 3 L of ethyl acetate at 40 °C for 5 × 8 h using a Soxhlet apparatus (CV Ruchi) [9]. The extract was concentrated on a rotatory evaporator (Buchi® brand) at approximately 40 °C until 55.7 g of a concentrated ethyl acetate extract of *M. pendans* was obtained.

### Separation and purification of the ethyl acetate extract *of M. pendans* bulbs

The concentrated ethyl acetate extract of *M. pendans* bulbs was separated by liquid column chromatography with stationary G_60_ silica gel (70–230 mesh) and 10% (*v*/v) of n-hexane-ethyl acetate as the eluent, and 11 fractions were produced. The contents of each fraction were then analysed using thin-layer chromatography (TLC) [10] with a stationary silica gel phase G_60_ F_254_ in determining the suitable solvent composition at subsequent purification process. The TLC results showed that fraction 3 of the first column chromatography step had relatively simple pattern of spots (R_f_ = 0.87) compared to other fractions. The fraction (5.7 g) was further purified using liquid column chromatography with a stationary G_60_ (70–230 mesh) silica gel phase and 2.5% (*v*/v) n-hexane-ethyl acetate as the eluent, and 17 fractions were produced. The fractions of the 2nd column chromatography step were analysed by TLC with a stationary silica gel (G_60_ F_254_) phase. The patterns of spots for 3-(7–9) showed a high number of individual components for 3-(7–9) to be considered pure (62.8 mg). The pattern of spots was further analysed by reversed-phase TLC using an octadecyl silane (ODS) stationary phase and 100% methanol as the eluent to determine a suitable solvent for the further purification of these fractions. The results of the analysis of fractions 3-(7–9) showed they had a constituent with the same R_f_ (0.62) as well as one other constituent. Then, the fraction was purified again by reversed-phase column chromatography using ODS RP-18 as the stationary phase and methanol with a 5% gradient of water (*v*/v) as the eluent, resulting in 8 fractions. The fractions of the 3rd column chromatography step were analysed using TLC with an ODS stationary phase, and this analysis showed the presence of a single compound, **compound 1**.

### Structural determination

The structure of **compound 1** was determined by analysing its ^1^H NMR, ^13^C NMR, HMQC, DEPT 135°, ^1^H-^1^H COSY and HMBC spectra, which were acquired on a 500 MHz FT-NMR spectrometer (ECA 500 JOEL variant, Japan). We used Delta™ NMR processing and control software, copyright 1990–2004 by JEOL USA, Inc. Version: 4.3.2 [Windows_NT] Network port = 6422. The IR spectrum of the compound was determined on an FT-IR Perkin Elmer Spectrum One spectrometer (Buckinghamshire, UK).

The number of carbon signals in **compound 1**, hybridization of the carbons (*sp*^3^, *sp*^2^, and *sp*), and the electronic environments impacting the chemical shift of each carbon atom in the compound were determined based on its ^13^C NMR spectrum. Information about the coupling of the signal from each carbon was also obtained from the ^13^C NMR spectrum using a DEPT parameter at 135°. The signals of the methine and methyl carbon atoms appeared as positive signals, whilst the signals of the methylene carbons appeared as negative signals. Signals from the quaternary carbons do not appear in DEPT spectra. The 2D NMR data were obtained by the DEPT 135° technique and from HMQC measurements. The HMQC spectrum showed the correlation data, or the relationships between protons and carbons one bond removed (^1^*J*). Determination of proton-to-proton coupling (^1^H-^1^H) across 3 or 4 bonds was performed by analysis of the ^1^H-^1^H COSY spectrum. The correlation of protons to protons in the ^1^H-^1^H COSY spectrum as indicated by the cross-peaks between the protons that are the result of spin matching.

The proposed structure of **compound 1** was confirmed by mass spectrometry (ES-MS Spectrometer, UPLC Type MS/MS TQD, Waters). The mass spectrum was acquired in the negative ion (ES-) mode, which means that the peaks observed will indicate molecular weights slightly lower than the actual molecular weights. Furthermore, the chemical shifts of **compound 1** were compared with those of a reference. The diterpenoid structure of **compound 1** was confirmed by comparison of the chemical shifts of **compound 1** with those of isocupressic acid, and the aliphatic chain was confirmed by the HMBC data of **compound 1**, and together, these data allowed us to assign all the chemical shifts. The results of the analysis of the two-dimensional NMR data confirmed the fragments and proposed structure of **compound 1**.

### Bacterial strain and inocula preparation

The bacteria used was *Streptococcus mutans* ATCC 25175, and the bacteria were streaked on Muller Hinton agar (MHA) and incubated at 37 °C for 48 h under facultative anaerobic conditions (5% CO_2_). For the inoculum preparation, one inoculating loop of bacteria was grown in liquid MHA medium overnight at 37 °C and adjusted to the appropriate optical density (at 595 nm) using a UV-VIS (Shimazu® brand) spectrophotometer. The bacterial suspension was then diluted until it reached the McFarland standard of 0.5 or it contained approximately 10^8^ CFU/mL [11].

### Determination of the minimum inhibitory concentration (MIC)

The MIC of **compound 1** from the bulbs of *M. pendans* was determined using a series of broth microdilutions according to the procedure described by Eloff [12] with a slight modification. **Compound 1** was serially diluted in a 96-well microplate with MH broth as the solvent until a 1–100 ppm concentration was obtained. One hundred microliters of inoculum (bacterial suspension) was added to the well, and then the plate was sealed with parafilm and incubated for 24 h at 37 °C. The MIC values were examined after the addition of 50 μL of crystal violet and incubation for an additional 30 min at 37 °C. The presence of bacterial growth was determined based on the colour change of the suspension in the well, and a reduction in the intensity of the violet colour indicated the activity of **compound 1** (terpenoid) [13, 14].

### Analysis of the minimum biofilm inhibitory concentration (MBIC)

The effect of this terpenoid from *M. pendans* bulbs on *S. mutans* biofilm formation was measured using a modified Perumal method [15]. Each well of a 96-well microplate was filled with brain heart infusion (BHI) and 1% sucrose medium between 20 μL and 160 μL of bacterial culture with a cell density of 10^8^ CFU/mL. To each well, 20 μL of terpenoid compound was added to give final concentrations of the compound from 1 to 100 ppm. Blank bacterial culture was used as a negative control, and the addition of 0.2% chlorhexidine was used as a positive control. After incubation for 24 h, the culture and the test compounds were removed, and each well was twice washed with 200 μL of phosphate-buffered saline (PBS). Afterwards, staining was performed by the addition of up to 200 μL of crystal violet stain and incubation for 30 min. The excess crystal violet stain was removed by rinsing three times with as much as 200 μL of PBS. Finally, up to 200 μL of 10% glacial acetic acid was added to each well to dissolve the crystal violet attached to the biofilm. Then, the absorbance was measured at a wavelength of 595 nm using a microplate reader.

### Minimum biofilm eradication concentration (MBEC)

The ability of the terpenoid from *M. pendans* bulbs to eradicate *S. mutans* biofilm was analysed using microdilution with a modified version of the method described by LaPlante [16]. The process of *S. mutans* biofilm production was as described for MBIC analysis. After biofilm production, **compound 1** (a terpenoid) was added, and the samples were left to stand for between 1 and 30 min. Wells containing bacteria were used as the negative control, and chlorhexidine was used as the positive control. The MBEC value was the lowest terpenoid concentration that was able to eradicate the biofilm, and the value was determined from the change in the colour of the well from the crystal violet and the MIC value.

## Results

### Thin-layer chromatography analysis

**Fig. 1:**
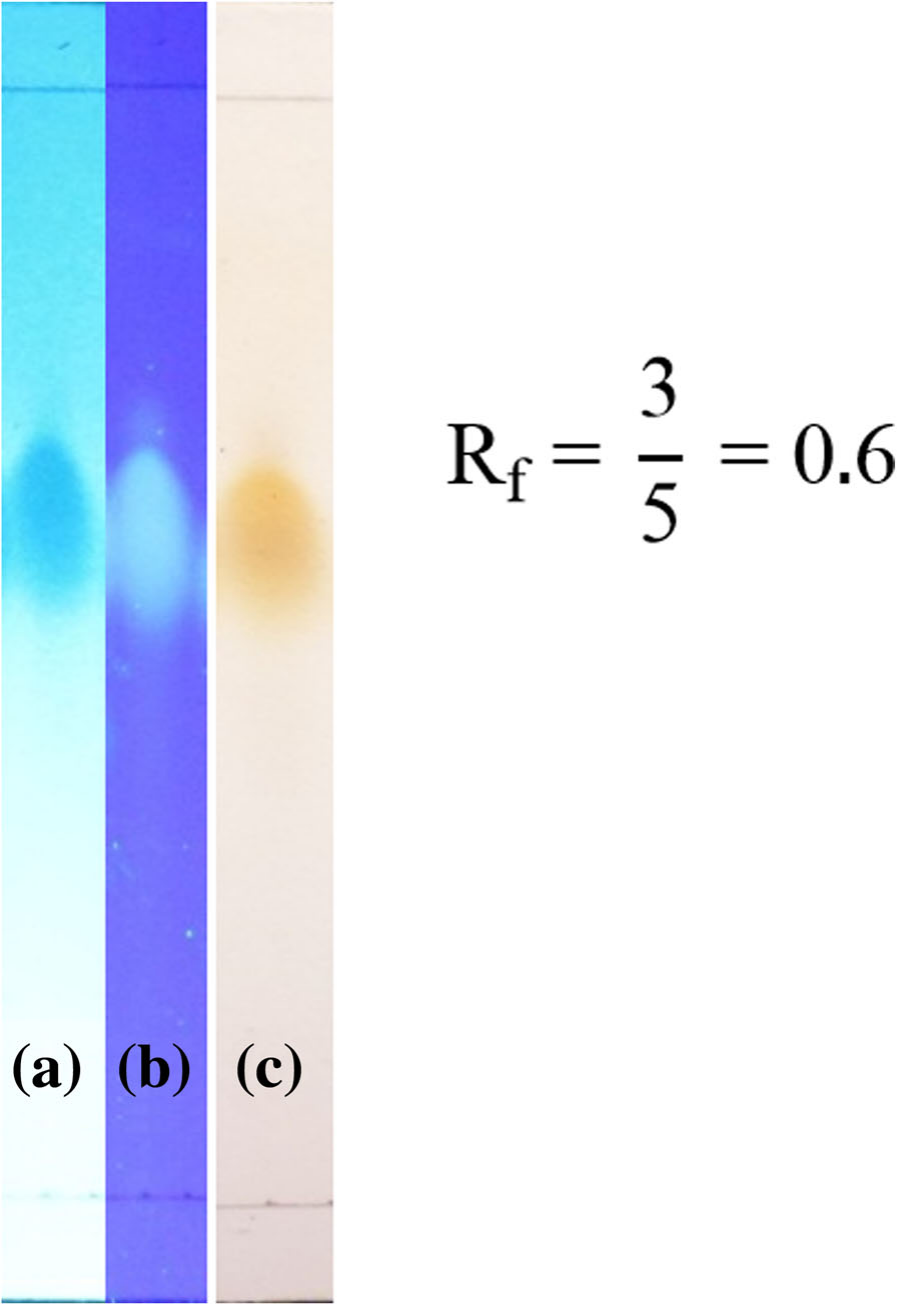
TLC chromatogram of fraction 3-(7–9)-(7&8) using an ODS plate with 100% methanol under UV light at λ 254 nm (**a**); UV light at λ 365 nm (**b**); and after spraying with 10% H_2_SO_4_ in methanol to make the colour persist (**c**)

The pattern of spots from the TLC of fractions 3-(7–9)- (7&8) from the third column chromatography purification with 100% methanol as the solvent is shown in Fig. 1. The fraction was found to have a component with an Rf = 0.6. This compound was subsequently named **compound 1**, and 33.2 mg of the compound was isolated.

### NMR analysis

**Compound 1** was a yellow, methanol-soluble oil that fluoresced under UV light (254 nm and 365 nm). These characteristics showed that there were n → π* and π → π * transitions available in **compound 1**. The transition of n → π* indicated that **compound 1** had a carbonyl group, and the transition of π → π* indicated that **compound 1** had a double bond. The results of the analysis of the ^1^H NMR, ^13^C NMR, HMQC, DEPT 135°, ^1^H-^1^H COSY, and HMBC spectra are shown in Table 1.

**Table 1:** NMR data of compound 1 (500 MHz for ^1^H NMR and 125 MHz for ^13^C NMR, CD_3_OD)

C Position	^13^C NMRδC (ppm)	DEPT 135°	^1^H –NMR δ_H_ (Int., mult., J = Hz)	HMBC ^1^H - ^13^C	COSY ^1^H - ^1^H
1	39.42	CH_2_	2.58 (2H)	–	–
2	27.0	CH_2_	1.29 (2H;*s*)	–	–
3	78.99	CH	3.19 (1H; *t; J* = 6.8)	–	–
4	44.5	Cq	–	–	–
5	35.45	CH		C-1, C-20	–
6	30.21	CH_2_	1.29 (2H;*s*)	–	–
7	40.57	CH_2_	2.41 (2H)	–	–
8	146.84	Cq	–	–	–
9	49.67	CH	1.98 (1H)	–	–
10	45.89	CH	1.98 (1H)	–	–
11	118.45	CH	4.85 (1H; *t*)	C-23, C-24	–
12	133.02	Cq	–	–	–
13	30.87	Cq	–	**–**	**–**
14	136.55	Cq	–	–	–
15	104.65	Cq	–	–	–
16	22.27	CH_2_	1.65 (2H; *d; J* = 9.1)	–	–
17	123.66	CH	4.92 (1H; *t*)	–	–
18	110.52	CH_2_	4.59 (1H; *s*); 4.37 (1H; *s*)	C-16	–
19	14.64	CH_3_	0.99 (3H; *d; J* = 3.9)	C-2, C-6	–
20	177.10	Cq	–	–	–
21	114.56	CH_2_	4.49 (2H; *s*)	C-8	–
22	18.00	CH_3_	1.48 (3H; *s*)	–	–
23	19.91	CH_3_	1.25 (3H, *s*)	C-14, C-27	–
24	17.11	CH_3_	1.52 (3H; *d; J* = 6.5)	C-12, C-22	–
25	12.96	CH_3_	1.25 (3H; *d; J* = 7.15)	C-27	H-26, H-27
26	34.19	CH_2_	1.98 (2H; *m*)	–	H-25
27	42.71	CH	2.56 (1H; *m*)	C-25	H-25
28	36.41	CH_2_	1.79 (2H; *t; J* = 7.48)	C-30	H-29
29	29.04	CH_2_	1.25 (2H; *d; J* = 7.15)	–	H-28
30	22.70	CH_3_	1.65 (3H; *d; J* = 9.1)	–	–
31	26.06	CH_3_	1.65 (3H; *d; J* = 9.1)	C-16	–

^13^C NMR: was used to determine the number of carbon signals.

HMQC: was used to determine the number of proton signals

DEPT 135°: was used to determine the number of methyl signals

COSY: was used to examine the correlations between hydrogens

HMBC: was used to determine the positions of functional groups and partial structures

The infrared spectrum (IR) of **compound 1** from a KBr pellet showed the presence of an absorption band at 3427 cm^− 1^, which is indicative of a hydroxyl group [17, 18]. The presence of the absorption at 2935 cm^− 1^ indicated the presence of an *sp*^3^ C-H bond. Additionally, there was a band at 1715 cm^− 1^ characteristic of a C=O (carbonyl) moiety [18]. There were also absorptions indicative of the gem dimethyl fragment at 1384 cm^− 1^ and 1450 cm^− 1^. The presence of the C-O bond in **compound 1** was indicated by an absorption band at 1172 cm^− 1^.

The ^13^C NMR spectrum of **compound 1** showed 31 carbon signals consisting of 22 *sp*^3^ carbon signals from δ_C_ 12.9–78.9 ppm, 8 *sp*^2^ carbon signals, and 1 carbonyl moiety (C=O) at δ_C_ 177.1 ppm. The peak with a shift of 78.9 ppm was thought to be from an oxygenated carbon [19].

The DEPT-135° spectrum of **compound 1** (**Fig. 4.7**) showed that **compound 1** had 7 methyl (CH_3_) signals at δC 12.9, 14.6, 17.1, 18.0, 19.9, 22.7, and 26.0 ppm; 10 methylene (CH_2_) signals composed of 8 *sp*^3^ methylene carbons (δ_C_ 22.2, 27.0, 29.0, 30.2, 34.1, 36.4, 39.4, and 40.5 ppm) and 2 *sp*^2^ methylene carbons (δ_C_ 110.5 and 114.5 ppm); and 7 methine (CH) signals comprising 4 *sp*^3^ methine carbons (δ_C_ 35.4, 42.7, 45.8 and 49.7 ppm), 1 oxygenated methyl carbon at δ_C_ 78.9 ppm and 2 *sp*^2^ methyl carbons at δ_C_ 118.4 and 123.6 ppm. There were also 7 quaternary carbons (Cq) comprising two *sp*^3^ quaternary carbon at δC 30.8 and 44.5 ppm, 4 *sp*^2^ quaternary carbons (δ_C_ 104.6, 133.0, 136.5, and 146.8 ppm), and 1 carbonyl quaternary carbon at δ_C_ 177.1 ppm.

The HMQC spectrum confirmed the numbers carbons and hydrogens determined from the ^13^C NMR and ^1^H NMR spectra as well as the environmental information determined from the proton signals. There was a one-bond correlation between H-22 (3H; 1.48 ppm) and C-22 (18.0 ppm) in **compound 1**, and these signals can be attributed to a methyl group. This correlation was supported by the DEPT-135° data, which showed a positive signal for C-22. Additionally, the binding of 3 protons confirmed that C-22 was a methyl group and not a methine group. A one-bond correlation was also observed from H-2 (2H, 1.29 ppm) to C-2 (27.0 ppm) and from H-6 (2H, 1.29 ppm) to C-6 (30.2 ppm), which were methylene groups. The one-bond correlations of H-21 (2H, 4.49 ppm) with C-21 (114.56 ppm) and H-18 (1H, 4.59 ppm; 1H, 4.37 ppm) with C-18 (110.52 ppm) indicated that **compound 1** contained an *sp*^2^ methylene carbon.

The ^1^H NMR spectrum showed the number, type, and environment of each of the protons in the compound. The protons in **compound 1** consisted of 42 protons in the region characteristic of protons attached to *sp*^3^ carbons, 1 proton with a chemical shift suggesting it was part of a hydroxy moiety, 1 proton in the chemical shifting region characteristic of protons attached to oxygenated *sp*^3^ carbons, and 6 protons in the region characteristic of protons attached to *sp*^2^ hybridized carbons. Protons in the *sp*^2^ proton chemical shift region were olefinic protons at δ_H_ 4.54 ppm. Many overlapping signals were observed for the protons in the *sp*^3^ proton chemical shift region, such as from δ_H_ 1.23–1.29 and δ_H_ 1.44–1.56 ppm. This overlap was caused by the similar chemical shifts in the *sp*^3^ region; thus, the ^1^H NMR spectrum in this region was difficult to interpret without any supporting data from other experiments such as two-dimensional NMR. The overlapping signals in the δ_H_ 1.23–1.56 ppm region were attributed to protons attached to the terpenoid carbon chains [20, 21].

The three-bond correlation between the proton at C-25 and the proton at C-26 confirmed that the protons were in the same spin system, and the correlation between the proton at C-28 and the proton at C-29 proved they were in the same system as well. These correlations showed that these protons were adjacent. The HMBC correlations were used to determine the positions of the functional groups and the partial structure of **compound 1**, and the HMBC spectrum showed ^*2*^*J–*^*3*^*J* correlations between protons and carbons.

The HMBC spectrum of **compound 1** showed correlations between the H-19 protons and C-2 and C-6. It can be assumed that there was a methyl group (C-19) attached at C-4 of the A ring. There were 2 *sp*^2^ methylene groups (C-18 and C-21) in **compound 1**. The ^*3*^*J* correlation between the H-18 protons and C-16 and the ^*2*^*J* correlation between the H-21 protons and C-8 helped elucidate the position of the double bond in **compound 1**. The ^*3*^*J* correlations between the H-14 protons and C-23 and between the H-24 protons and C-22 suggested a gem dimethyl group was present at the C-13 position. The presence of a gem dimethyl fragment was supported by the absorption bands at 1384.90 and 1450.96 cm^− 1^ in the infrared spectrum. There were also 2 double bonds in **compound 1** (δ_C_ 123.66 ppm, 136.55 ppm, 146.84 ppm, and 118.45 ppm). The double bond between C-11 and C-14 was confirmed by the correlations between H-11 and C-24, H-11 and C-23, and H-14 and C-23. The ^*3*^*J* correlations between H-25 and C-27 and between H-27 and C-25 indicated the second double bond was between C-25 and C-26.

**Fig. 2:**
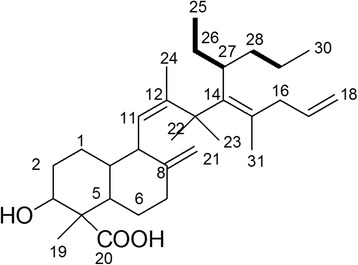
Proposed structure of compound 1

Based on the connectivity of the fragments of **compound 1** determined from analysis of the two-dimensional NMR data, the structure of **compound 1** was proposed to be that of a diterpenoid derivative. This compound has a molecular formula of C_31_H_50_O_3_ with seven degrees of unsaturation. The proposed structure of **compound 1** is shown in Fig. 2.

Based on the results of the mass spectrometric measurements, **compound 1** had a molecular weight of *m/z* 469.99. This result supported our proposed structure of **compound 1**, which has a molecular formula of C_31_H_50_O_3_ and a molecular weight of 470.

Based on a comparison of the NMR data of **compound 1** to those of a reference compound, it was clear that **compound 1** has signals that similar to those of the reference compound; thus, **compound 1** was determined to be a diterpenoid derivative [22]. Furthermore, **compound 1** was a **terpenoid**.

### Inhibition of *S. mutans* biofilm formation by a terpenoid from *M. pendans* bulbs

The MIC value of the isolated terpenoid towards *S. mutans* was 40 ppm. The MBIC value of the terpenoid towards *S. mutans* biofilm formation was 50 ppm (Table 2). However, with three repetitions, the standard deviation for each measurement increased. This result was probably caused by the tendency of *S. mutans* to aggregate, which altered the amount of bacteria present in the initial culture preparation in each batch, which in turn impacted each measurement.

**Table 2:** Inhibition and Eradication of by a terpenoid from *Myrmecodia pendans* against *Streptococcus mutans* biofilm

Bacteria	MIC (ppm)	MBIC (ppm)	MBEC %
*S. mutans*	40	50	40%

### Results of the ability of the terpenoid from *M. pendans* to eradicate *S. mutans* biofilm

The MBEC value of the terpenoid towards the *Streptococcus mutans* biofilm for 1 min of treatment tended to increase; thus, the eradication percentage, or the amount of biofilm damaged also increased over time. At concentrations above 100 ppm, the relationship between activity and concentration was no longer linear, and those data were not included in the graph. The terpenoid from *M. pendans* bulbs was able to eradicate approximately 40% of the biofilms that had formed in 1 min at the highest concentrations tested (Table 2). After 30 min of treatment, a discrepancy was found between different experiments. Only one plate showed results consistent with those found for the 1 min treatment.

## Discussion

The results of the NMR experiments conducted in this study showed that **compound 1** was a diterpenoid derivative called a **terpenoid** with a molecular formula of C_31_H_50_O_3_. Previous research conducted by Widyawati [23] also found an active compound, which was a diterpenoid derivative, with the molecular formula of C_25_H_40_O_4_. However, there was a different substituent present at C11, and the number of carbons was different (25 carbons in the prior study compared to 31 carbons in this study). Although the core of the compounds was the same, the overall characteristics showed that they were different compounds. The MIC value of the C_25_H_40_O_4_ terpenoid towards *S. mutans* was 78.125 ppm [23], while the MIC of the terpenoid isolated in this study was 40 ppm. Perumal’s study [15] showed that the most active compound of *Euphorbia hirta L.* was a terpenoid. Terpenoids are the largest group of natural plant products known for having potential antimicrobial activities.

The MBIC value of the terpenoid from *M. pendans* bulbs towards *S. mutans* biofilm was 50 ppm, and its MIC value was 40 ppm. The concentration of a compound required to inhibit the growth of a bacterial biofilm was higher than that required for a planktonic biofilm. This is because the exopolysaccharides contained in the biofilms make penetration more difficult [24]. The active compounds of the natural substances show bactericidal and bacteriostatic effects by preventing bacterial attachment to the surface of the pharynx, skin, and tooth mucosa; inhibition of glycolytic enzymes; pH reduction; reduction of biofilm and plaque formation; and reduction of the hydrophobicity of the cell surface [25].

In this study, when the highest concentrations of the *M. pendans* terpenoid were tested for 1 min, they could destroy approximately 40% of the biofilms that had been formed. Cowan [26] stated that the target of active compounds for bacterial eradication could be reached through disruption of the mechanisms of cell wall biosynthesis and permeability of the cell membranes, surface adsorption of compound components, inhibition of protein synthesis or nucleic acid metabolism, or inhibition of the enzyme activity through oxidation. Terpenoids can influence the release of planktonic cells from biofilms. Terpenoids can also influence the membrane integrity of all organisms and eradicated most biofilm cells [27].

This study was the first that successfully showed the antimicrobial effect of a terpenoid from *M. pendans* under both the planktonic conditions and against *S. mutans* biofilms. Thus, natural compounds can be considered potential molecules for the prevention of dental plaque [28]. Natural substances were shown to have a direct effect on the formation of cariogenic biofilms by inhibiting the expression of glucosyltransferase activity in *S. mutans* and *S. sobrinus* [3, 29].

## Conclusions

A terpenoid with a molecular formula of C_31_H_50_O_3_ extracted from *M. pendans* has potential to be developed as an antibacterial agent*,* particularly with to prevent the formation of biofilms with an MBIC value of 50 ppm and to eradicate approximately 40% of *S. mutans* biofilms.

## Abbreviations

ATCC: American type culture collection
CFU: Colony forming unit
COSY: Correlation spectroscopy
DEPT: Distortionless enhancement by polarization transfer
ES-MS: Electrospray mass spectrometry
HMBC: Heteronuclear multiple bond correlation
HMQC: Heteronuclear multiple quantum coherence
IR: Infrared
NMR: Nuclear magnetic resonance
TLC: Thin-layer chromatography
UPLC: Ultra-performance liquid chromatography
UV-VIS: Ultraviolet-visible

## References

[1] Klein M, Duarte S, Xiao J, Mitra S, Foster T, Koo H (2009). Structural and molecular basis of the role of starch and sucrose in *Streptococcus mutans* biofilm development. Applied and Environmental Microbiology.

[2] Yoshida A, Kuramitsu H (2002). Multiple *Streptococcus mutans* genes are involved in biofilm formation. Appl Environ Microbiol.

[3] Jeon J, Rosalen P, Falsetta M, Koo H (2011). Natural products in caries reasearch: current (limited) knowledge, challenges and future perspective. Caries Res.

[4] Vrani E, La-Evi A, Mehmedagi A, Uzunovi A (2004). Formulation ingredients for toothpastes and mouthwashes. Bosnian Journal of Basic Medical Sciences.

[5] Gold J (2008). The role of chlorhexidine in caries prevention. Oper Dent.

[6] Supriatno DR (2014). Antitumor activity of Papua’s *Myrmecodia pendens* in human oral tongue squamous cell carcinoma cell line through induction of cyclin-dependent kinase inhibitor p27Kp1 and suppression of cyclin E. J of Cancer Res and Ther.

[7] Soeksmanto A, Subroto M, Wijaya H, Simanjuntak P (2010). Anticancer activity test for extracts of sarang semut (*Myrmecodia pendens*) to HeLa and MCM-B2 cells. Pak J Biol Sci.

[8] Soeksmanto A, Simanjuntak P, Subroto MA (2012). Uji toksisitas akut ekstrak air tanaman sarang semut (*Myrmecodia pendans*) terhadap histologi organ hati mencit. Jurnal Nature Indonesia.

[9] Abdolshahi A, Mojteba HH, Jared SR, Mehrdad T, Aliakbar S, Jaime ATS (2015). Choice of solvent extraction technique affects fatty acid composition of pistachio (Pistacia vera L.) oil. J Food Sci Technol.

[10] Kustrin SA, David WM, Ahmad PY (2013). Thin-layer chromatography-bioassay as powerful tool for rapid identification of bioactive compounds in botanical extracts. Mod Chem Appl.

[11] Gartika M, Inne SS, Mieke HS, Alex C, Danny H (2014). Antibacterial activities of papain against *Streptococcus mutans* ATCC 25175. Int Journal of Development Research.

[12] Eloff JN (1998). A sensitive and quick microplate method to determine the minimal inhibitory concentration of plant extracts for bacteria. Plant Med.

[13] Gupta A (2015). Biofilm quantification and comparative analysis of MIC (minimum inhibitory concentration) and MBIC (minimum biofilm inhibitory concentration) value for different antibiotics against E. Coli. Int J Curr Microbiol App Sci.

[14] Gordya N, Andrey Y, Anastasia K, et al. Natural antimicrobial peptide complexes in the fighting of antibiotic resistant biofilms: *Calliphora vicina* medicinal maggots. Plos One. 2017;12(3) 10.1371/journal.pone.0173559 PMC5344439 28278280

[15] Perumal S, Roziahanim M (2013). Chemical analysis, inhibition of biofilm formation and biofilm eradication potential of *Euphorbia hirta* L. against clinical isolates and standard strains. BMC Complementary & Alternative Medicine.

[16] LaPlante KL, Sarkisian SA, Woodmansee S, Rowley DC, Seeram NP (2012). Effects of cranberry extracts on growth and biofilm production of Escherichia coli and Staphylococcus species. Phytother Res.

[17] Patra JK, Kim ES, Oh K, Kim HJ, Dhakal R, Kim Y, Baek KH (2015). Bactericidal effect of extract and metabolites of *Robinia pseudoacacia* L. on *Streptococcus mutans* and *Porphyromonas gingivalis* causing dental plaque and periodontal inflammatory diseases. Molecules.

[18] Dong WW, Jiao W, Deng MC, Yang CB, Yue JM, Lu RH (2008). A new steroid glycoside derivative from Acorus calamus L. J of The Chinese Chemical Society.

[19] Khatun M, Billah M, Quader MA (2012). Sterols and sterol glucoside from Phyllanthus species. Dhaka Univ. J. Sci.

[20] Seebacher W, Simic N, Weis R, Saf R, Kunert O. Complete assignments of 1H and 13*C NMR* resonances of oleanolic acid, 18α-oleanolic acid, ursolic acid and their 11-oxo derivatives. Magn Reson Chem. 41:636–8. 10.1002/mrc.1214.

[21] Govenalp Z, Kilik N, Kazaz C, Kaya Y, Demiezer LO (2006). Chemical constituen of Galium tirtunerte. Turk J.

[22] Filho ER, Magnani RF, Xie W, Mirocha CJ, Pathre SJ (2002). Hidroxiliation of labdane diterpene cupresic acid by *Fusarum graminearum*. J Braz Chem.

[23] Widyawati. Potential of terpenoid isolated from Myrmecodia pendans as antibacterial against Streptococcus mutans. Int Journal of Development. Research. 2016; 6(10):10350–4.

[24] Suzana KS, Robert EWH (1758). Mode of action the new antibiotic for gram positive pathogens daptomycin comparison with cationic antimicrobial peptides and lipeptides. Biochim Biophys Acta.

[25] Abachi S, Song L, Rupaisinghe HPV (2016). Molecular mechanism of inhibition of Streptococcus species by phytochemicals. Molecules.

[26] Cowan MM (1999). Plant product as antimicrobial agents. Clin Microbiol Rev.

[27] Mastelic J, Politeo O, Jerkovic I, Radosevic N (2005). Composition and antimicrobial activity of *Helichrysum italicum* essential oil and its terpene and terpenoid fractions. Chem Nat Comp.

[28] Sa NC, et al. Antimicrobial and antibiofilm action of Carbane diterpene from *Croton nepetaefolius* against oral bacteria. SciVerse ScienceDirect, 2011. 10.1016/j.archoralbio.2011.10.016. 10.1016/j.archoralbio.2011.10.016 22119044

[29] Kalesinskas P, Kačergius T, Ambrozaitis A, Peciuliene V, Ericson D (2014). Reducing dental plaque formation and caries development, a review of current methods and implications for novel pharmaceuticals. Stomatologija, Baltic Dental and Maxillofacial Journal.

